# Synthesis and Biological Evaluation of Novel Isoxazole-Amide Analogues as Anticancer and Antioxidant Agents

**DOI:** 10.1155/2021/6633297

**Published:** 2021-03-09

**Authors:** Ahmad M. Eid, Mohammed Hawash, Johnny Amer, Abdullah Jarrar, Samira Qadri, Iman Alnimer, Aya Sharaf, Raya Zalmoot, Osama Hammoudie, Saba Hameedi, Ahmed Mousa

**Affiliations:** ^1^Department of Pharmacy, Faculty of Medicine and Health Sciences, An-Najah National University, Nablus P.O. Box 7, 00970 Palestine, State of Palestine; ^2^Department of Biomedical Sciences, Physiology, Pharmacology & Toxicology Division, Faculty of Medicine and Health Sciences, An-Najah National University, Nablus, Palestine, State of Palestine

## Abstract

Cancer now is one of the leading causes of mortality in the world. There has been a lot of effort to discover new anticarcinogenic agents that allow treatment with fewer side effects. A series of isoxazole-carboxamide derivatives (**2a**–**2g**) were synthesised and evaluated for their cytotoxic activity against breast (MCF-7), cervical (HeLa), and liver (Hep3B) cancer cell lines and their antioxidant activity in the 2,2-diphenyl-1-picrylhydrazyl (DPPH) assay. The results showed that **2d** and **2e** were the most active compounds against Hep3B cells, with a half-maximal inhibitory concentration (IC_50_) of around 23 *μ*g/ml; **2d** showed the highest activity against HeLa cells, with an IC_50_ 15.48 *μ*g/ml. However, **2a** had the lowest IC_50_ (39.80 *μ*g/ml) against MCF-7 cells. By contrast, compound **2**g was inactive against all cancer cell lines, with IC_50_ values >400 *μ*g/ml. Both **2d** and **2e** reduced Hep3B secretion of alpha-fetoprotein (to 1829.33 ± 65.91 ng/ml and 1758.66 ± 54.04 ng/ml, respectively). Furthermore, in cell cycle analysis, **2d** and **2e** induced a delay in the G2/M phase of 18.07%, which is similar to the doxorubicin positive control. Moreover, **2d** and **2e** reduced the necrosis rate of Hep3B threefold and instead shifted the cells to apoptosis. Our results indicate that **2d** and **2e** have potent and promising anticancer activity. However, compound **2a** was the most active as antioxidant agent (IC_50_ = 7.8 ± 1.21 *μ*g/ml) compared with Trolox as a positive control (IC_50_ 2.75 *μ*g/ml).

## 1. Introduction

Cancer is the leading cause of death throughout the world [1–3]. Indeed, 25% of the deaths in developing countries are due to cancer [4–6]. In 2018, cancer was responsible for 9.6 million deaths. During a person's life, 1 out of 5 men and 1 out of 6 women will develop cancer [7]. Environmental factors such as diet, obesity, alcohol consumption, physical inactivity, radiation, sunlight, and viral infection as well as genetic factors lead to the development of cancer [8]. Over the years, scientists have explored a myriad of treatments for each type of cancer, including chemotherapy, hormonal treatment, radiation, and surgery [9].

**Figure 1:**
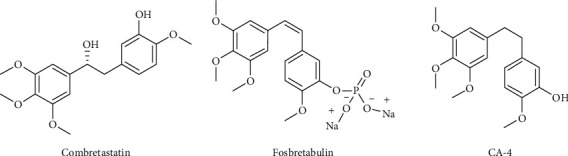
Combretastatin, CA-4P (fosbretabulin), and CA-4 structures.

Chemotherapy has been widely used, particularly against inoperable cancer [10], as the primary therapy or as an adjunct therapy before and/or after another treatment [11]. However, chemotherapy use is restricted because it has weak effectiveness, minimal selectivity against target cells, and undesirable side effects such as alopecia, queasiness, and vomiting [12, 13]. Many natural extracts with anticancer activity have been evaluated in recent decades [14]. One of these compounds, combretastatin (Figure 1), was isolated from the African plant *Combretum caffrum* and has been modified to find new derivatives with antitumour activity, such as combretastatin A-4 phosphate (fosbretabulin) (Figure 1). That compound has been approved by the Food and Drug Administration (FDA) for thyroid cancer [15–17].

**Figure 2:**
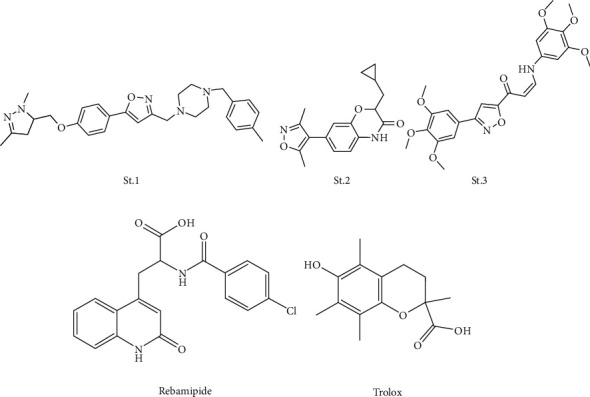
Isoxazole containing compounds with anticancer activities, and antioxidant agents (Rebamipide and Trolox).

Different types of heterocyclic derivatives such as isoxazole are used extensively as agrochemicals in medicine; indeed, they are efficient in anticancer chemotherapy [15, 18]. Researchers have found that the isoxazole ring imparts it with anticancer [19, 20], hypoglycemic [21], pain killing, bactericidal [20, 22], antiviral (for HIV) [23], and anti-inflammatory [24] activities. The isoxazole ring has been used as a core structure for many anticancer medications [25]. For example, resorcinylic 4,5-diarylisoxazole amides have shown a potent inhibitory effect on heat shock protein (HSP90) [26]. A derivative of diarylisoxazole (Figure 2, st. 1) was discovered for its activity against androgen receptor- (AR-) expressing breast cancer cells [27]. Leflunomide, an immunosuppressant used in chemotherapy, is another isoxazole derivative. Other researchers reported that the 3,5-dimethylisoxazole (Figure 2, st. 2) functional group mimics acetylated lysine (KAc); they utilised this functional group to improve bromodomain inhibitors, with positive effects on cancer cells [28]. In another work, researchers tested a hybrid molecule, with a trimethoxyphenyl moiety combined with the isoxazole ring (arylamino-isoxazolyl-2-propenone; Figure 2, st. 3). The compound showed cytotoxic activity (low half-maximal inhibitory concentration (IC_50_) values) against several cancer cell lines: cervical adenocarcinoma (HeLa), lung adenocarcinoma (A549), breast carcinoma (MCF7), and hepatocellular carcinoma (HCT116) [29].

Oxidants are formed as a normal product of aerobic metabolism, but they can be produced at elevated rates under pathophysiological conditions. Thus, an imbalance between oxidants and antioxidants in favour of oxidants potentially leads to damage that forms the core of oxidative stress. The biologically active agents that work to slow or prevent the cell damage caused by those free radicals are called antioxidants [30, 31]. Environmental stress is usually the primary impetus for the formation of these unstable free radicals. While the human body produces endogenous antioxidants, other agents are found in natural plants and foods; examples include *β*-carotene, R-tocopherol (vitamin E), and ascorbic acid (vitamin C). Other antioxidants can be chemically synthesised [30–33]. In the last few decades, researchers have synthesised many agents that have marked antioxidant activity, such as quinolinone-3-aminoamide [31], thienopyrimidine, thienopyrazole [34], and *N*-aryl-1,4-dihydropyridine derivatives [35]. These substances, like Trolox, a water-soluble vitamin E analogue, and rebamipide (Figure 2), exhibit effective antioxidant properties by scavenging unstable free radicals [31, 36, 37].

The current project is aimed at synthesising novel isoxazole-carboxamide derivatives (**2a**–**2g**) with or without a methoxyphenyl moiety and at evaluating some of their biological activity such as antioxidant and anticancer activities on HeLa, MCF-7, HepG2, and HepB3 cancer cell lines.

## 2. Materials and Methods

### 2.1. Chemistry

All chemicals were obtained from Alfa Aesar and Sigma-Aldrich. SMP-II Digital Melting Point Appliances are used to determined melting points and are uncorrected. ^13^C-NMR and ^1^H-NMR spectra were recorded in DMSO-d6 and were performed on two NMR instruments. The first was a Bruker 500J MHz-Avance III High-Performance Digital FT-NMR spectrometer at the Faculty of Science, Department of Chemistry, the University of Jordan, Jordan. The second was a Bruker 300 MHz-Avance III High-Performance Digital FT-NMR spectrometer at the NMR facility at the Doping and Narcotics Analysis Laboratory of the Faculty of Pharmacy, Anadolu University, Turkey. Tetramethylsilane was used as the internal standard. All chemical shifts were recorded as d (ppm). High-resolution mass spectra data (HRMS) were collected using a Waters LCT Premier XE Mass Spectrometer (high sensitivity orthogonal acceleration time-of-flight instrument) using the ESI (+) method (the instrument was coupled to an AQUITY Ultra Performance Liquid Chromatography system (Waters Corporation, Milford, MA, USA)) at Pharmacy Faculty, Gazi University, Ankara, Turkey.

#### 2.1.1. General Procedure of Isoxazole-Carboxamide Synthesis (**2a**–**2g**)

3-(2-Chlorophenyl)-5-methylisoxazole-4-carboxylic acid (1) (1.5 mmol) was dissolved in dichloromethane (12 ml). To this mixture DMAP (0.3 mmol), EDC (1.8 mmol) were added and was allowed to stir under nitrogen gas at room temperature for 30 min. After that, the appropriate aniline derivative (1.8 mmol) was added and the mixture was allowed to stir for 24-48 h. The reaction was monitored by TLC, and at the end of the reaction, the solvent was removed under reduced pressure and dissolved again in dichloromethane, then extracted with 1% NaHCO_3_ and brine. The organic layer was dried by drying agent Na_2_SO_4_ and evaporated under reduced pressure. The obtained product was purified by flash chromatography using the appropriate solvent system or by the crystallization utilising appropriate solvent system [38].

#### 2.1.2. N-(4-(tert-butyl)phenyl)-3-(2-chlorophenyl)-5-methylisoxazole-4-carboxamide (**2a**)

Silica gel column was purified by chromatography using n-hexane: ethyl acetate solvent system (70 : 30); solid product, M.P. 175-176°C, yield 77%; ^1^H NMR (DMSO-d6) *δ*: 10.02 (1H, s), 7.43-7.58 (6H, m), 7.31 (2H, d, *J* = 8.6 Hz), 2.65 (3H, s), and 1.24 (9H, s); ^13^C NMR (DMSO-d_6_) *δ* ppm: 169.98, 160.40, 159.71, 146.74, 136.57, 132.84, 132.24, 131.97, 130.09, 127.98, 127.79, 125.78, 120.04, 115.13, 34.50, 31.54, 12.73. HRMS (*m*/*z*): [M+H]^+^ calcd for C_21_H_22_ClN_2_O_2_ 369.1356, found 369.1364.

#### 2.1.3. N-(4-chloro-2,5-dimethoxyphenyl)-3-(2-chlorophenyl)-5-methylisoxazole-4-carboxamide (**2b**)

Purified by silica gel column chromatography using n-hexane: ethyl acetate solvent system (70 : 30); solid product, M.P. 115-117°C, yield 67%; ^1^H NMR (DMSO-d_6_) *δ*: 8.83 (1H, s), 8.02 (1H, s), 7.55-7.74 (4H, m), 7.07 (1H, s), 3.75 (3H, s), 3.52 (3H, s), and 2.75 (3H, s); HRMS (*m*/*z*): [M+H]^+^ calcd for C_19_H_17_Cl_2_N_2_O_4_ 407.0565, found 407.0558.

#### 2.1.4. 3-(2-chlorophenyl)-N-(3,5-dimethoxyphenyl)-5-methylisoxazole-4-carboxamide (**2c**)

Purified by silica gel column chromatography using n-hexane: ethyl acetate solvent system (65 : 35); solid product, M.P. 255-257°C, yield 82%; ^1^H NMR (DMSO-d_6_) *δ*: 9.99 (1H, s), 7.45-7.59 (4H, m), 7.24 (1H, d, *J* = 2.1 Hz), 7.02 (1H, dd, *J* = 8.7, 2.1 Hz), 6.87 (1H, d, *J* = 8.7 Hz), 3.70 (3H, s), 3.69 (3H, s), and 2.65 (3H, s); ^13^C NMR (DMSO-d_6_) *δ* ppm: 169.89, 160.44, 159.45, 148.94, 145.76, 132.83, 132.70, 132.23, 131.99, 130.11, 127.99, 127.82, 112.41, 112.19, 105.26, 56.19, 55.81, and 12.79; HRMS (*m*/*z*): [M+H]^+^ calcd for C_19_H_18_ClN_2_O_4_ 373.0955, found 373.0951.

#### 2.1.5. 3-(2-Chlorophenyl)-N-(3,4-dimethoxyphenyl)-5-methylisoxazole-4-carboxamide (**2d**)

Purified by silica gel column chromatography using n-hexane: ethyl acetate solvent system (75 : 25); solid product, M.P. 199-200.5°C, yield 90%; ^1^H NMR (DMSO-d_6_) *δ*: 9.99 (1H, s), 7.47-7.59 (4H, m), 7.23 (1H, d, *J* = 2.1 Hz), 7.02 (1H, dd, *J* = 8.4, 2.1 Hz), 6.87 (1H, d, *J* = 8.7 Hz), 3.70 (3H, s), 3.68 (3H, s), and 2.65 (3H, s); HRMS (*m*/*z*): [M+H]^+^ calcd for C_19_H_18_ClN_2_O_4_ 373.0955, found 373.0948.

#### 2.1.6. 3-(2-Chlorophenyl)-N-(2,5-dimethoxyphenyl)-5-methylisoxazole-4-carboxamide (**2e**)

Purified by silica gel column chromatography using n-hexane: ethyl acetate solvent system (65 : 35); solid product, M.P. 188-189°C, yield 78%. ^1^H NMR (DMSO-d_6_) *δ*: 8.27 (1H, s), 7.79 (1H, s), 7.58-7.68 (4H, m), 6.86 (1H, d, *J* = 9 Hz), 6.06 (1H, dd, *J* = 9, 3 Hz), 3.67 (3H, s), 3.48 (3H, s), and 2.75 (3H, s).^13^C NMR (DMSO-d_6_) *δ* ppm: 173.86, 158.94, 153.35, 143.18, 135.35, 133.35, 132.79, 132.52, 130.51, 128.30, 127.77, 127.22, 111.94, 108.76, 107.43, 56.55, 55.85, and 13.17; HRMS (*m*/*z*): [M+H]^+^ calcd for C_19_H_18_ClN_2_O_4_ 373.0955, found 373.0954.

#### 2.1.7. 3-(2-Chlorophenyl)-5-methyl-N-phenylisoxazole-4-carboxamide (**2f**)

Purified by silica gel column chromatography using n-hexane: ethyl acetate solvent system (70 : 30); solid product, M.P. 201-202°C, yield 69%. ^1^H NMR (DMSO-d_6_) *δ*: 10.14 (1H, s), 7.47-7.58 (6H, m), 7.30 (2H, t, *J* = 7.5 Hz), 7.07 (1H, t, *J* = 7.2 Hz), and 2.66 (3H, s). ^13^C NMR (DMSO-d_6_) *δ* ppm: 170.10, 160.45, 159.86, 139.13, 132.79, 132.25, 132.03, 130.10, 129.19, 127.90, 127.83, 124.35, 120.27, 115.06, and 12.77. HRMS (*m*/*z*): [M+H]^+^ calcd for C_17_H_14_ClN_2_O_2_ 313.0744, found 313.0748.

#### 2.1.8. 3-(2-Chlorophenyl)-5-methyl-N-(3,4,5-trimethoxyphenyl)isoxazole-4-carboxamide (**2g**)

Purified by silica gel column chromatography using n-hexane: ethyl acetate solvent system (70 : 30); solid product, M.P. 214-215.5°C, yield 90%; ^1^H NMR (DMSO-d_6_) *δ*: 10.06 (1H, s), 7.47-7.60 (4H, m), 6.93 (2H, s), 3.71 (6H, s), 3.61 (3H, s), and 2.66 (3H, s). ^13^C NMR (DMSO-d_6_) *δ* ppm: 170.03, 160.42, 159.68, 153.19, 135.33, 134.31, 132.85, 132.19, 131.99, 130.12, 127.98, 127.83, 115.07, 97.93, 60.57, 56.21, and 12.80. HRMS (*m*/*z*): [M+H]^+^ calcd for C_20_H_20_ClN_2_O_5_ 403.1048, found 403.1055.

### 2.2. Biological Methods

#### 2.2.1. Cell Culture and Cytotoxicity Assay

Hep3B, HeLa, and MCF7 cancer cell lines were cultured in Roswell Park Memorial Institute- (RPMI-) 1640 medium and supplemented with 10% foetal bovine serum, 1% l-glutamine, and 1% penicillin/streptomycin in a humidified atmosphere with 5% CO_2_ at 37°C. The cells were seeded at 2.6 × 10^4^ cells/well in a 96-well plate. After 72 h, the cells were confluent; the medium was changed and cells were incubated with various concentrations (500, 100, 50, 10, and 1 *μ*g/ml) of the synthesised compounds for 24 h [14]. Cell viability was assessed with the Cell Tilter 96® Aqueous One Solution Cell Proliferation (MTS) assay according to the manufacturer's instructions (Promega Corporation, Madison, WI). Briefly, at the end of the treatment, 20 *μ*l of MTS solution per 100 *μ*l of medium was added to each well and incubated at 37°C for 2 h. The absorbance was measured at 490 nm [39, 40].

#### 2.2.2. Alpha-Fetoprotein (aFP) Analysis

Hep3B cells were cultured in Dulbecco's Modified Eagle Medium (DMEM) with 10% foetal bovine to examine the level of aFP as a marker of tumour activity. We marked Hep3B cells by labelling their surface with HBsAg (Water et al., 1998). Hep3B cells were incubated with DDW and silica in 10 *μ*l/ml for 24 h. A commercially available enzyme-linked immunosorbent assay (ELISA) kit (R&D Systems, Inc., Minneapolis, MN, USA) was used to assess the level of aFP in the medium.

#### 2.2.3. Apoptosis and Cell Cycle Analysis

Hep3B cells were trypsinised (0.05% trypsin/0.53 mM EDTA), washed, and adjusted to 1 × 10^6^ cells/ml (in saline containing 1% albumin; Biological Industries, Israel) for 10 min to determine their purity. The cells were fixed with 4% paraformaldehyde and permeabilised in 0.1% saponin in phosphate-buffered saline (PBS) for 20 min at room temperature. They were then stained with an anti-human HBsAg monoclonal antibody (R&D System) for 30 min at room temperature. The cells were then incubated with propidium iodide (PI) to stain fragmented DNA and Annexin V conjugated to fluorescein isothiocyanate (FITC) (R&D systems) to stain phosphatidylserine (PS) according to the manufacturer's instructions. The cells were analysed by flow cytometry (Becton-Dickinson LSR 11, Immunofluorometry Systems, Mountain View, CA, USA). Apoptotic cells were defined as Annexin V (+)/PI (-). Viable cells were defined as Annexin V (-)/PI (-). In each experimental setting, unstained controls, immunoglobulin G (IgG) isotype controls, and FMO controls were used [41].

To analyse the cell cycle, Hep3B was fixed in cold 70% ethanol for at least 30 min at 4°C. After washing two time in PBS, the cells were treated with 50 *μ*l RNase (100 *μ*g/ml), stained with 5 *μ*l propidium iodide (PI) (50 *μ*g PI/100 ml solution), and analysed with flow cytometry [41].

### 2.3. Antioxidant Activity

The antioxidant activity was measured for synthesised compounds **2a**–**2g**. A 1 mg/ml solution of each compound was prepared by dissolving 1 mg in 1 ml of methanol; this stock was diluted with methanol to obtain several concentrations (2, 5, 10, 20, 50, and 100 *μ*g/ml). One millilitre of each concentration was mixed with 1 ml of methanol and 1 ml of 2,2-diphenyl-1-picrylhydrazyl (DPPH) solution. The solution was incubated for 30 min in the dark at room temperature. A blank solution was prepared by replacing the plant fraction with methanol [42]. Trolox was used as a positive control. The absorbance was measured by a UV-Vis spectrophotometer at 517 nm then compared with the control. The antioxidant activity was calculated with the following equation:

(1) $$\begin{array}{c} \text{I}\left(\%\right)=\left.\left[{\text{ABS}}_{\text{blank}}–\text{AB}{\text{S}}_{\text{test}}\right]/\left[{\text{ABS}}_{\text{blank}}\right]\right)\times 100\%, \end{array}$$

where I (%) is the percent antioxidant activity and ABS is the absorbance at 517 nm.

The antioxidant IC_50_ for each synthesised compounds was calculated using BioDataFit 1.02 (data fit for biologists) [43].

### 2.4. Statistical Analyses

The antioxidant activity was measured in triplicate for each sample, while the cytotoxic test was measured in duplicate for each sample. The obtained results are presented as the mean ± standard deviation. Statistical analysis was performed using the GraphPad Prism software version 6.01 (GraphPad Software, San Diego, CA, USA). Three or more groups were compared with one-way analysis of variance (ANOVA) followed by Bonferroni's post hoc test.

## 3. Results and Discussion

### 3.1. Chemistry

**Figure 3:**
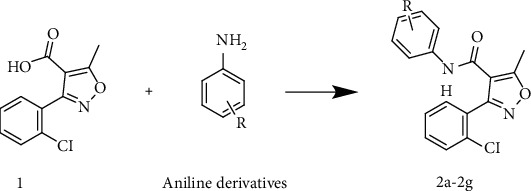
1+ aniline derivatives dissolved in 15 ml DCM, then DMAP and EDC added under nitrogen gaz stir for 24 h.

The isoxazole-carboxamide derivatives (**2a**–**2g**) were synthesised as outlined in Figure 3. The coupling to form the isoxazole-carboxamide compounds **2a**–**2g** was afforded by using EDC as an activating agent and DMAP as a covalent nucleophilic catalyst; the active intermediates were reacted with the aniline derivatives [44]. High-resolution mass spectrometry (HRMS) was used to confirm the synthesis of these seven compounds. They were purified by using column chromatography (a 70 : 30 mixture of *n*-hexane : ethyl acetate solvent). The proton nuclear magnetic resonance (^1^H-NMR) peaks confirmed the synthesis of these compounds. There was one proton in the range of 8.27–10.14 ppm as a singlet peak for the N-H amide of each compound; there were 6–9 protons (depending on whether or not the phenyl was substituted) in the aromatic area and another 3 protons around 2.66 ppm as a singlet peak, which refers to the methyl group on the isoxazole ring. The ^13^C-NMR spectrum showed a C signal of a carbonyl group around 170 ppm; a signal at 12–13 ppm indicated an aliphatic carbon methyl.

### 3.2. Biological Evaluations

#### 3.2.1. Cytotoxic Evaluation of the Compounds **2a**–**2g**

Table 1 shows the cytotoxic effect of compounds **2a**–**2g** on MCF-7, HeLa, and Hep3B cancer cell lines. Compounds **2d** and **2e** showed the greatest anticancer activity against Hep3B cells, while the rest of the compounds had a high IC_50_; a compound with an IC_50_ > 100 *μ*g/ml is listed as “inactive” in Table 1. Compound **2d** was the most potent against HeLa cells (IC_50_ = 18.62 *μ*g/ml), and compound **2a** was also active against HeLa cells (IC_50_ = 39.80 *μ*g/ml). However, the other compounds showed weak anticancer activity against MCF-7 cells (IC_50_ values from 63.10 to 588.80 *μ*g/ml).

#### 3.2.2. Alpha-Fetoprotein Results

**Figure 4:**
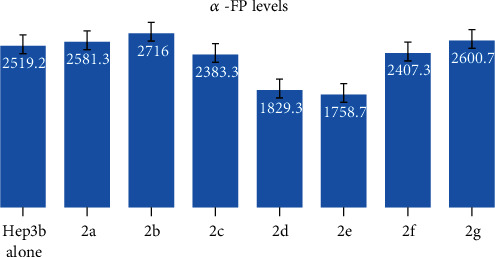
Cell proliferations of synthesized compound **2a**–**2g** and untreated cells (control).

According to cytotoxicity results and because **2d** and **2e** were the most active against Hep3B cells, we evaluated the levels of aFP secreted into the medium to examine the inhibitory effects of the synthesised compound on cell proliferation. Compounds **2d** and **2e** reduced Hep3B secretion of aFP to 1829.33 ± 65.91 and 1758.66 ± 54.04 ng/ml, respectively, compared with 2519.17 ± 198.05 ng/ml in untreated cells. Compounds **2c** and **2f** did not reduce aFP secretion (2383.33 and 2407.33 ng/ml, respectively) compared with **2d** and **2e** (Figure 4). Overall, **2d** and **2e** have anticancer activity against Hep3B cells (Figure 4).

#### 3.2.3. **2d** and **2e** Inhibited DNA Cell Cycle of Hep3B Cells

**Figure 5:**
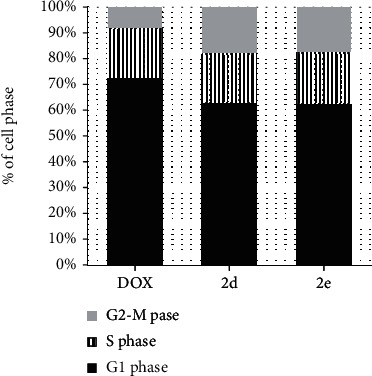
Cell cycle analysis of Hep3B cells after treatment with compound **2e**, **2d**, and DOX control.

Because **2d** and **2e** were cytotoxic to Hep3B cells, they were assessed to determine whether they disturbed the cell cycle. Doxorubicin (DOX) was used to a positive control to induce cell cycle progression. The data in Figure 5 show a similar proportion of cells in the G1 phase following treatment with compounds **2d** or **2e** or DOX. Compounds **2d** and **2e** also showed similar behaviour to DOX in reducing the percent of cells in the S as well as the G2/M phase (*P* < 0.05). Indeed, **2d** and **2e** reduced the proportion of cells in the G2/M phase up to 3.6-fold. These data indicate that **2d** and **2e** markedly delay mitosis, suggesting they have potential anticancer characteristics.

#### 3.2.4. Apoptosis vs. Necrosis Test

**Figure 6:**
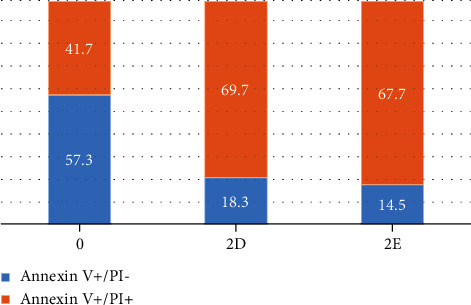
Apoptosis vs. necrosis for compounds **2d** and **2e**.

We next determined whether **2d** and **2e** induced apoptosis (programmed cell death) and necrosis. Cells undergoing apoptosis have their PS phospholipids translocated from the inner face of the plasma membrane to the cell surface. Therefore, apoptotic cells can be identified by the presence of PS on the cell surface using Annexin V-FITC staining and flow cytometric analysis. Cells were also stained with PI, which can enter the cell only when the plasma membrane is damaged. Apoptosis was evaluated as Annexin V (+)/PI (-); this population was distinguished from late apoptotic and necrotic cells, which were considered Annexin V (+)/PI (-). Untreated Hep3B cells showed 57.33% ± 7.5% apoptotic cells; treatment with **2d** and **2e** reduced apoptosis to 14.53% ± 3.4% and 18.26% ± 2.1%, respectively (Figure 6). Treatment with **2d** and **2e** increased the Annexin V (+)/PI (+) fraction (apoptotic/necrotic cells) to 67.66% ± 4.9% and 69.67% ± 3.3%, respectively, compared with 41.66% ± 1.52% in untreated cells. Our data strongly suggest that compounds **2d** and **2e** have anticancer properties through increasing the necrotic activity of hepatocellular cancer cells and thus accelerating their death.

### 3.3. Antioxidant Evaluation

**Figure 7:**
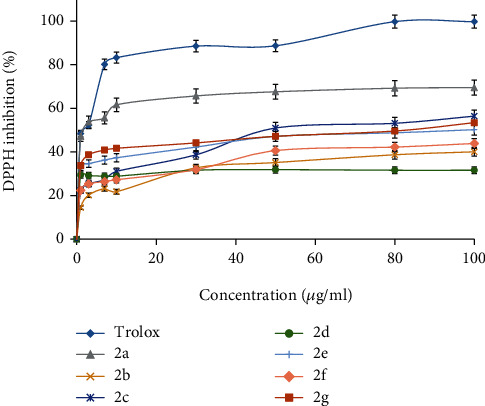
The DPPH inhibition percentage of synthesized compounds and Trolox.

We estimated the antioxidant activity of compounds **2a**–**2g** using an *in vitro* DPPH assay; we compared the results to the well-known antioxidant compound Trolox. We tested a range of concentrations to determine the IC_50_ of each compound. The most active compound was **2a**, with an IC_50_7.8 ± 1.21 *μ*g/ml, while **2c**, **2e**, and **2g** had an IC_50_ of 56.1, 67.6, and 51.2 *μ*g/ml, respectively. For comparison, the Trolox IC_50_ was 2.75 *μ*g/ml. Compounds **2b**, **2d**, and **2f** had very weak antioxidant activity (Figure 7).

One of the main causes of diseases such as cancer, stroke, inflammatory bowel disease, atherosclerosis, and cirrhosis is thought to be oxidative stress, which is caused by free radicals [45]. The genomic integrity of the cell is maintained by a balance between the levels of prooxidants and antioxidants. The host's immunity is modulated if this balance is destructed and will affect the normal cellular signaling pathways leading to uncontrolled proliferation of the cells, ending with cancer and macrophage polarisation leading to the formation of atherogenic plaques [46, 47]. In these conditions, particularly in the tumour microenvironment, there is higher basal oxidative stress that takes advantage of the upregulated antioxidant system [23].

## 4. Conclusion

The synthesised compounds showed moderate to high activity against Hep3B, HeLa, and MCF-7 cancer cell lines compared with the commonly used anticancer drug DOX. Of note, compounds **2d** and **2e** showed superiority compared with the other synthesised compounds. Moreover, compound **2a** showed great potential to ameliorate oxidative stress compared with the standard Trolox, followed by compounds **2g**, **2c**, and **2e**. Future plans could involve docking and synthesis of additional analogues of this core structure to examine the structure–activity relationship.

## Figures and Tables

**Table 1:** IC_50_ of isoxazole compounds and doxorubicin on different cancer cells (Hep3B, HeLa, and MCF-7).

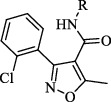		IC_50_ (*μ*g/ml)		
Code	R Group	Hep 3B	Hela	MCF
2a	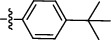	79.43 ± 2.34	18.62 ± 0.79	39.80 ± 1.63
2b	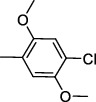	Inactive	45.70 ± 1.67	85.10 ± 2.72
2c	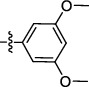	Inactive	Inactive	75.80 ± 2.36
2d	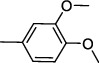	23.98 ± 1.83	15.48 ± 0.89	Inactive
2e	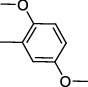	23.44 ± 1.99	32.35 ± 3.05	63.10 ± 2.14
2f	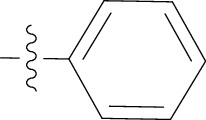	81.28 ± 2.23	Inactive	98.5 ± 2.57
2g	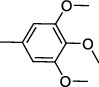	Inactive	Inactive	Inactive
DOX		1.25 ± 0.19	2.13 ± 0.07	1.55 ± 0.23

Note: *P* value ≤ 0.05.

## Data Availability

The datasets used and/or analysed during the current study available from the corresponding author on reasonable request.
